# Epidemiological and molecular investigation of resurgent cutaneous leishmaniasis in Sudan

**DOI:** 10.1016/j.ijid.2019.08.018

**Published:** 2019-11

**Authors:** Sarah Collis, Sayda El-Safi, Atia A. Atia, Tapan Bhattacharyya, Awad Hammad, Margriet Den Boer, Hai Le, James A. Whitworth, Michael A. Miles

**Affiliations:** a London School of Hygiene and Tropical Medicine, London, UK; b Faculty of Medicine, University of Khartoum, Sudan; c World Health Organization, Sudan; d KalaCORE Consortium, Sudan; e Médecins Sans Frontières, Amsterdam, The Netherlands; f KalaCORE Consortium, London, UK

**Keywords:** Sudan, *Leishmania major*, Cutaneous leishmaniasis, Epidemiology

## Abstract

**Objectives:**

Local health personnel have drawn attention to an apparent increase in incidence and severity of cutaneous leishmaniasis (CL) in Sudan. The objective of this study was to investigate CL burden and surveillance.

**Methods:**

Surveillance data were compiled from the KalaCORE programme, *Leishmania* coordinators in Northern Kordofan and Southern Darfur, and Khartoum Dermatology Hospital. CL lesions were sampled from 14 suspected cases from Northern Kordofan and the Hospital for Tropical Diseases in Omdurman. PCR–restriction fragment length polymorphism analysis and multilocus sequencing were used to characterize the disease agent.

**Results:**

All sites reported substantial increases from 2014 to 2016/7, far exceeding World Health Organization case reports for 2014, consistent with a widespread outbreak. Single seasonal peak incidence was observed, except for two peaks in Southern Darfur. In Northern Kordofan, the odds ratio for CL in the 35–44 years age group was 2.6 times higher than in the >45 years age group (*p* < 0.0001); in Southern Darfur, the OR was 2.38 greater in males than females (*p* < 0.0001). Lesions included severe presentations, despite chemotherapy. *Leishmania major* was identified as the agent.

**Conclusions:**

Active surveillance is required to understand the extent of CL in Sudan, as well as training to standardize surveillance, diagnosis, reporting, and quality control. Point-of-care rapid diagnosis would be valuable. Genotyping and phenotyping are required to monitor the emergence of pathogenic strains, drug resistance, outbreaks, and changes in severity.

## Highlights

• A cutaneous leishmaniasis (CL) outbreak has occurred in Sudan.
• The number of cases far exceeds that stated in external (World Health Organization) reports.
• The clinical presentation is severe, despite chemotherapy.
• *Leishmania major* is the disease agent.
• Active surveillance, standardized reporting, and a rapid diagnostic test are required.

## Introduction

Cutaneous leishmaniasis (CL) is the most common form of the leishmaniases, which are caused by *Leishmania* protozoa. These are transmitted by female phlebotomine sand flies and principally affect impoverished and weakened populations. CL is endemic in 98 countries, with an estimated 0.6 million to 1 million new cases occurring annually (Alvar et al., 2012). The agents of CL in eastern Africa are principally *Leishmania tropica*, *Leishmania major*, and *Leishmania aethiopica*, with different clinical outcomes. In Sudan, *L. major*, of the strain group named zymodeme LON-1, is considered commonly responsible for CL; *L. tropica* is known from neighbouring countries such as Egypt, Kenya, and Ethiopia (World Health Organization, 2010). Life-threatening visceral leishmaniasis (VL) is due to *Leishmania donovani* in eastern Africa and *Leishmania infantum* along the Mediterranean coast.

CL can mimic many other skin conditions (World Health Organization, 2018a). Parasitological confirmation via microscopy remains the gold standard of CL diagnosis and is the most affordable approach, but relies heavily on parasite yield, sampling, quality of stained slides of skin biopsies, and the skills and experience of personnel (Reithinger et al., 2007, World Health Organization, 2014). Alternative molecular detection techniques on either skin or blood might offer greater sensitivity, but these are not widely available or standardized (de Vries et al., 2015, Vink et al., 2018, World Health Organization, 2014).

Although rarely fatal, the disease can leave substantial scarring and disfigurement, with stigmatization and social exclusion. In Africa, chemotherapy with pentavalent antimonials, such as the generic sodium stibogluconate (SSG) given intralesionally or intravenously, is only indicated for lesions lasting more than 6 months, multiple or large lesions, or lesions on a joint or the face (World Health Organization, 2010). Alternative treatments may be local paromomycin and methylbenzethonium or oral fluconazole (Burza et al., 2018, World Health Organization, 2010). Treatment costs and hospital visits can be a significant financial burden for families and public health resources.

The World Health Organization (WHO) Leishmaniasis Coordinator for Sudan recently reported an apparent increase in cases of CL with an unusually aggressive manifestation, atypical for the endemic *L. major*, and for which the disease agent had not been identified (unpublished observations, current authors SE-S and AAA). Thus, the purpose of this investigation was to review the epidemiological surveillance data and to characterize the disease agent.

## Methods

### Epidemiological data

All CL cases in the datasets analysed here were reported to the authors of this study as being parasitologically confirmed. Epidemiological data were supplied from the following sources:

1 The KalaCORE consortium (http://www.kalacore.org/), which supports the control and elimination of VL in Africa and Asia. KalaCORE concomitantly collected limited Sudanese CL incidence data for 2016. Data were analysed in Microsoft Excel. Sex, age, and treatment data were not available.
2 Khartoum Dermatology Hospital (KDH), a nationwide CL referral centre. KDH provided the data for 2012–2016 that were analysed here. It was unclear from which exact populations the cases originated, thus odds ratios (OR) for geographical origin were not calculated. Excel was used for analysis.
3 The ministries of health (MOHs) of Northern Kordofan and Southern Darfur states. The Northern Kordofan Health Information System (HIS) reported the incidence of CL for 2010 to June 2016. The Leishmania coordinator for Northern Kordofan provided line listing of parasite-confirmed CL patients who attended the two hospitals at El Obied and Um Ruwaba for July 2016 to July 2017, based on monthly reporting by healthcare professionals. Southern Darfur gave corresponding information for January 2016 to August 2017. Line listings on consultation date, age, and sex were translated into English, transferred to Excel, and the data cleaned manually. ORs for age and sex were derived in Stata 14 using projected population estimates for 2016 (Central Bureau of Statistics, Republic of Sudan). Values of *p* < 0.05 were considered significant.

Reports on surveillance, laboratory training, and treatment challenges for Northern Kordofan and for the Hospital for Tropical Diseases (HTD) in Omdurman, Khartoum, were consulted in collaboration with community and hospital teams.

### Clinical samples

All samples from lesions were collected by authorized local technical staff. A total of 14 suspected CL patients with active (not dry or healing) skin lesions were identified for analysis of *Leishmania spp*, as described below. Samples CL01–CL08 were collected in remote areas of Northern Kordofan. The remaining six samples (CL10–CL15) were taken at the HTD clinic. Samples for microscopic and PCR analysis were collected by pipette from slit skin smears, with four samples taken from each patient: (1) directly onto Whatman FTA card (WHAWB120210; Sigma-Aldrich, UK); (2) into ATL buffer (939011; Qiagen, UK); (3) into sterile vacuettes (454001; Greiner, UK) containing 2 ml of RPMI culture medium (R0883; Sigma), supplemented with 10% foetal calf serum (F9665; Sigma), penicillin–streptomycin (P4333; Sigma), and 5-fluorocytosine (F7129; Sigma); and (4) as a smear on slides, taken previously and examined by local personnel before this study for the presence of amastigotes.

### DNA extraction

DNA extraction From ATL buffer samples and cultures was performed using the QIAamp DNA Mini Kit (51304; Qiagen) according to the manufacturer’s instructions.

### *Leishmania* species identification and multilocus sequence typing (MLST)

*ITS*1-*Hae*III PCR–restriction fragment length polymorphism (PCR-RFLP) analysis was applied for species identification (Schönian et al., 2003); MLST was performed on genes previously established for *Leishmania* typing, encoding cytosolic NADP-malic enzyme (*ME*), fumarate hydratase (*FH*), and 6-phosphogluconate dehydrogenase (*PGD*) (Supplementary material, Table S1) (Zemanova et al., 2007). Purified MLST amplicons were sequenced bi-directionally and compared with genome sequences for *L. major* strains, accessed via www.TriTrypdb.org: Friedlin (MHOM/IL/81/Friedlin) (Ivens et al., 2005), LV39 (MRHO/SU/59/P) (Rogers et al., 2011), and SD75 (MHOM/SN/74/SD) (National Center for Biotechnology Information, 2012). These three strains originate from Israel, Uzbekistan, and Senegal, respectively.

## Results

### Epidemiological observations

**Figure 1:**
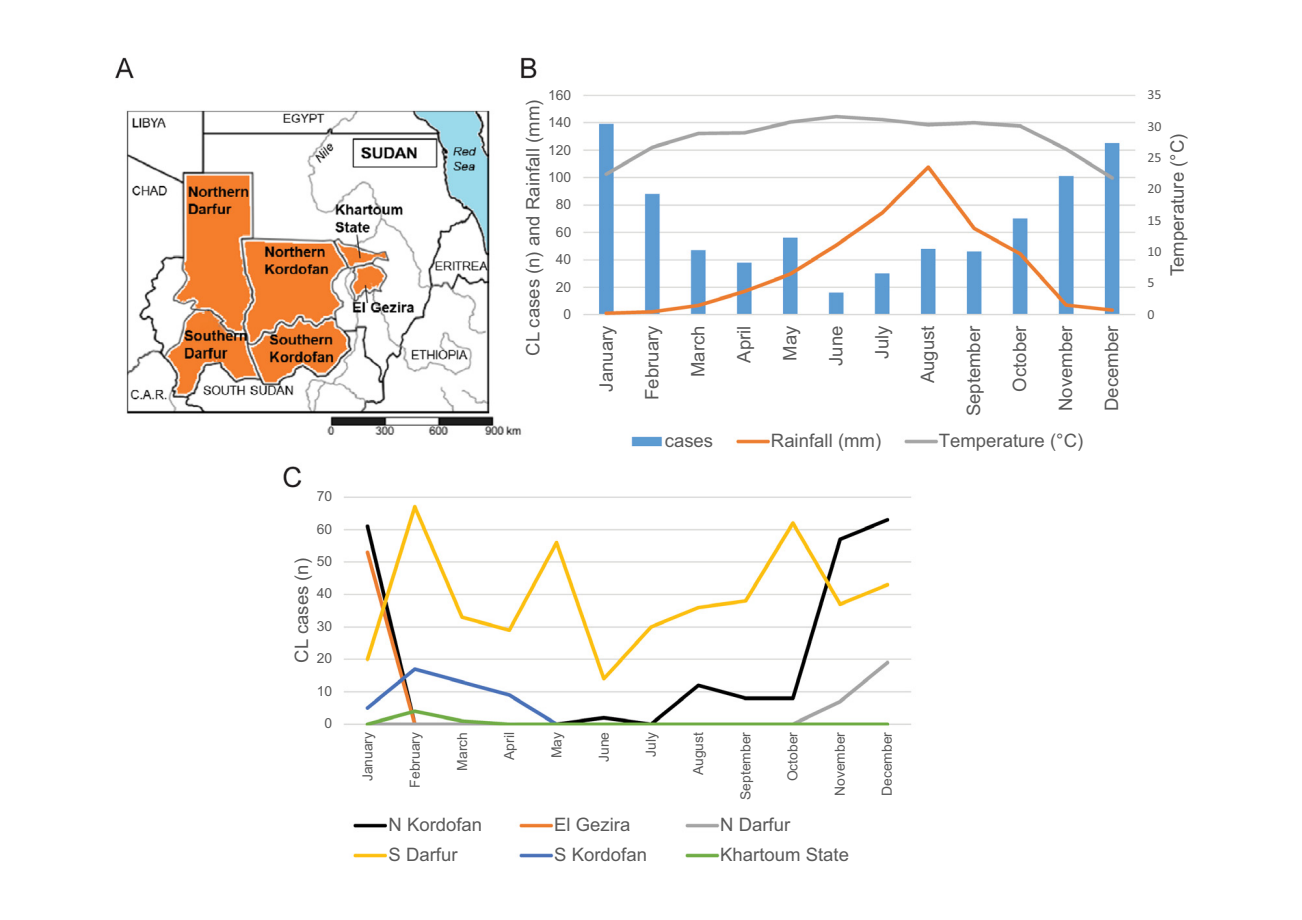
KalaCORE data. (A) Geographical sources of data. (B) KalaCORE cutaneous leishmaniasis data for 2016 (blue bars), with temperature and rainfall data for 2015 overlaid, indicating a decrease in case reports with rising rainfall. (C) KalaCORE individual state data showing that Southern Darfur had additional peaks of case reports in May and October.

Geographical sources of CL epidemiological data and clinical samples are shown in Figure 1A. KalaCORE nationwide CL case incidence data per month and by state in 2016, reported to the WHO Leishmania coordinator from each state’s MOH department, indicated a total of 804 new CL cases from Northern Darfur, Southern Darfur, Northern Kordofan, Southern Kordofan, El Gezira, and Khartoum states. The highest numbers of new cases were in the winter months (January, February, November, December) and the lowest in the summer months (Figure 1B). Separate analysis by state showed that Southern Darfur reported additional peaks in May and October (Figure 1C).

KDH reported a total of 3801 CL cases between 2012 and 2016 (Table 1). Compared to cases in 2013 (*n* = 416), the number of cases increased more than threefold in both 2014 (*n* = 1350) and 2015 (*n* = 1421), indicating an outbreak starting in 2014. The 1350 cases seen at KDH alone in 2014 was more than the 1053 cases that the WHO reported in the same year for the entire country (World Health Organization, 2018b). Overall, between 2012 and 2016 there were consistently more cases among males than females, cumulatively 2178 (57.3%) male cases compared to 1599 female cases; sex was not recorded for 24 cases (Table 1). Most cases were in the 15–24 years age group (*n* = 1080, 28.4%), followed by the 25–44 years age group (*n* = 974, 25.6%); the fewest cases were in infants less than 1 year of age (*n* = 72, 1.9%), followed by those older than 60 years (*n* = 136, 3.6%) (Table 1).

**Table 1:** Cutaneous leishmaniasis cases reported by Khartoum Dermatology Hospital 2010–2016.

		2012	2013	2014	2015	2016	Total
All cases	Number	206	416	1350	1421	408	3801 (100%)
Sex	Male	112	249	775	809	233	2178 (57.3%)
	Female	94	167	575	612	151	1599 (42.1%)
	Not recorded	–	–	–	–	24	24 (0.6%)
Age (years)	<1	7	8	25	26	6	72 (1.9%)
	1–4	37	33	118	126	30	344 (9.1%)
	5–14	41	88	341	341	96	907 (23.9%)
	15–24	48	146	367	391	128	1080 (28.4%)
	25–44	55	96	337	358	128	974 (25.6%)
	45–59	10	32	107	128	11	288 (7.6%)
	60+	8	13	55	51	9	136 (3.6%)

**Figure 2:**
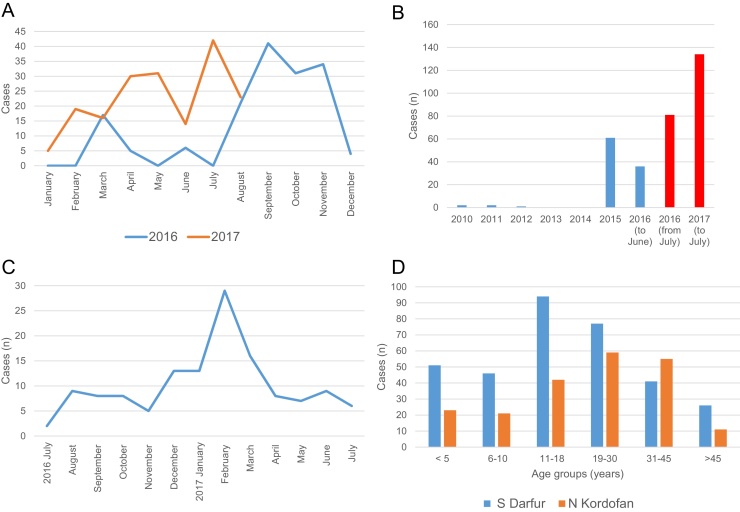
Southern Darfur and Northern Kordofan Ministry of Health data. (A) Southern Darfur Ministry of Health cutaneous leishmaniasis case reports showing differing profiles in 2016 (blue line) and 2017 (orange line). (B) Northern Kordofan cutaneous leishmaniasis cases reports by the Ministry of Health – Health Information System between 2010 and June 2016 (blue bars), and by hospital line listing from July 2016 to July 2017 (red bars), showing a sharp increase from 2015. (C) Northern Kordofan State cutaneous leishmaniasis case reports between July 2016 and July 2017, for which the month was reported, showing a peak in winter (February); 46 cases were excluded as the month was not reported. (D) Age distribution of cases, Southern Darfur (blue bars) and Northern Kordofan (orange bars).

Between January 2016 and August 2017, Southern Darfur MOH reported a total of 342 CL cases: 159 in 2016 and 180 in 2017; the year was not specified for three cases. The seasonal trend for 2017 cases was the opposite of that for 2016 cases (Figure 2A). In Northern Kordofan, MOH-HIS reported 102 cases between 2010 and June 2016, of which five were reported before 2015. Two hundred and fifteen CL cases were reported from the two hospital line listings between July 2016 and July 2017. In the first 6 months of 2017 alone, cases were more than double those in each of the previous 2 years reported by MOH-HIS (Figure 2B). The highest number of cases was reported in February 2017 (Figure 2C).

Data reported from Southern Darfur and Northern Kordofan MOHs included the age and sex distribution of CL cases, from which ORs were calculated. In Southern Darfur, age was reported for 335 of 342 cases. Of all cases, 50% were in the 11–18 years and 19–30 years age groups (Figure 2D), with the fewest cases in those >45 years of age. There was no significant difference in ORs between age groups. Of the 342 cases, 247 (72.2%) were male, and the OR of CL in the males was over twice that in the females (OR 2.38, 95% confidence interval (CI) 1.87–3.05, *p* < 0.0001) (Supplementary material, Table S2).

In Northern Kordofan, the majority of cases were in age groups 19–30 years (27.4%) and 31–45 years (25.6%), and the fewest in those >45 years of age (5.1%) (Figure 2D). The age group 35–44 years had 2.6 times the OR of CL when compared to those >45 years of age (*p* = 0.0004); in the 20–34 years group this was 2.1 times (*p* = 0.0030), but with no significant difference when compared to the 0–19 years age group (*p* = 0.6525) (Supplementary material, Table S2). Of the 215 cases, 108 (50.2%) were male and 105 (48.8%) were female (sex not reported in two cases), with an OR of CL in the male subjects slightly higher than that in female subjects (OR 1.11, 95% CI 0.84–1.47), but this was not statistically significant (*p* =  0.45) (Supplementary material, Table S2).

### Clinical presentation

**Figure 3:**
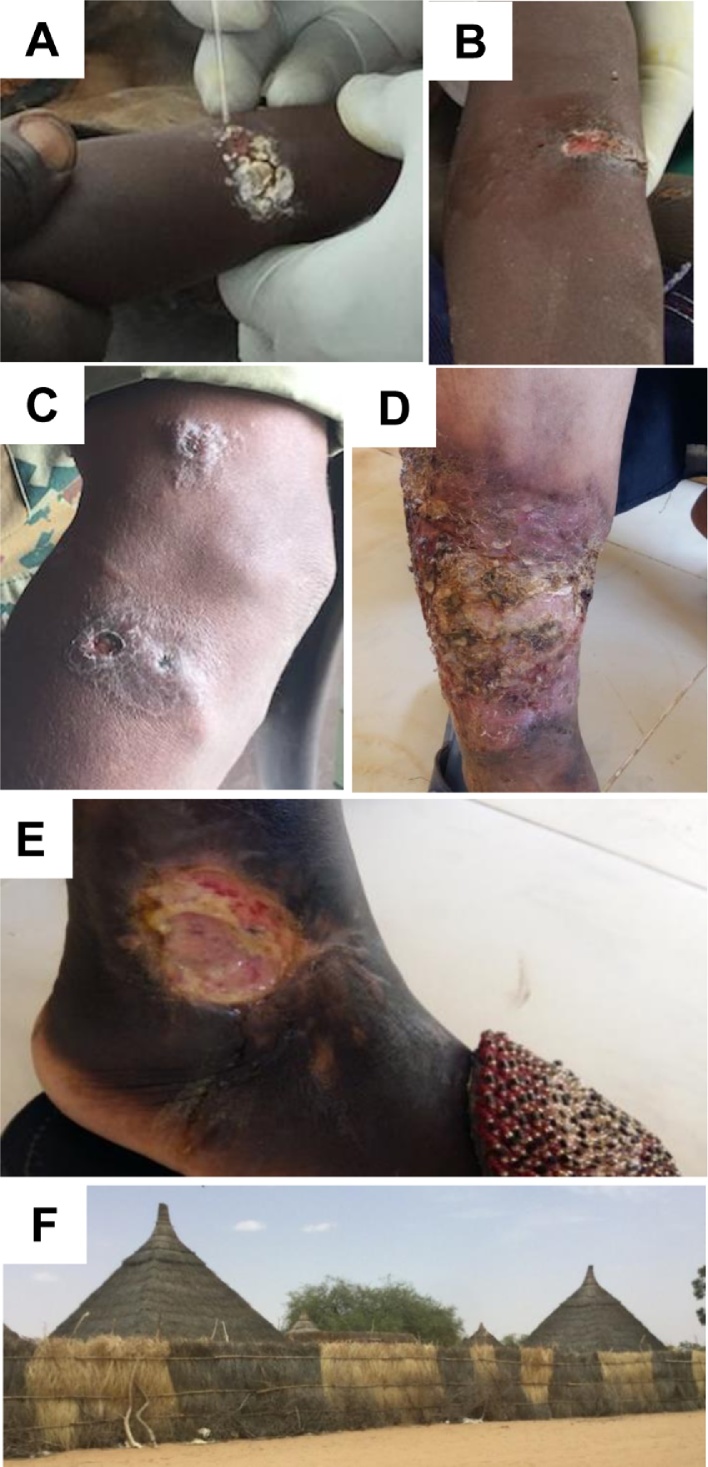
Cutaneous leishmaniasis lesions. (A)–(C) Northern Kordofan State: (A) CL05, (B) CL06, (C) CL08. (D) and (E) Hospital for Tropical Diseases, Omdurman: (D) CL11, 3-year-old lesion, reported as Pentostam-resistant with multiple secondary infections, (E) CL12, a 2-year-old cutaneous lesion on the ankle of a sickle cell anaemia patient, also not responding to treatment. (F) Straw huts in a remote farming village near Barrah, North Kordofan State. *Leishmania major* was identified in samples from this location (CL05 and CL06).

Of the 14 lesion samples collected, coded here as CL01–CL08 and CL10–CL15, four were from females and 10 were from males. The average age of individuals from whom lesions were sampled was 22 years (range 3–67 years). Four participants lived in huts made from tree branches, four in a mosque, two outside, three in a house, and one in a tent. Only one participant reported using a bed net. Two participants were classified as refugees. Ten participants reported the onset of symptoms between 7 days and 4 months previously. Four had chronic lesions (CL10, CL11, CL12, CL14), with onset of symptoms ranging from 1 to 4 years. All suspect lesions were located on limbs, except for patient CL10 who had nodular lesions on the nose and ear. The lesions ranged in size from 2.5 cm to 10 cm in diameter. Seven lesions were ulcerative, three nodular-ulcerative, and four nodular. At least three out of 14 participants (CL10, CL11, CL12) had received SSG treatment prior to sampling. Examples of lesions are shown in Figure 3A–E.

Samples CL01–CL08 were collected in remote areas of Northern Kordofan. Samples CL05 and CL06 originated from the same village near Barrah. The samples belonged to a 3-year-old female and 4-year-old male, respectively. Both lived in straw huts in a farming community and appeared visibly malnourished. CL05 had a single lesion that was ulcerative, painless, and approximately 3 cm in diameter, with symptoms beginning approximately 4 months earlier. CL06 had two ulcerative lesions that were painless and approximately 3 cm in diameter, with symptoms of the first lesion beginning approximately 2 months earlier. CL08, a 55-year-old male soldier who normally lived outside or in a tent in Northern and Southern Kordofan, had six nodular-ulcerative lesions, approximately 4 cm in diameter, situated on both his arms and legs. Symptoms began 2 months prior to sampling. The remaining six samples (CL10–CL15) were collected at the HTD clinic, where severe lesions were also seen that were 2 to 3 years old (CL11, CL12), previously parasitologically confirmed, and non-responsive to SSG treatment (Figure 3). At HTD, where CL lesions had been seen in patients with co-infections, an 18-year-old male from Eastern Sudan was admitted with both VL and active CL lesions on the hand and elbow, with urinary tract, malaria, and HIV co-infections.

### *Leishmania* species identification and characterization

**Figure 4:**
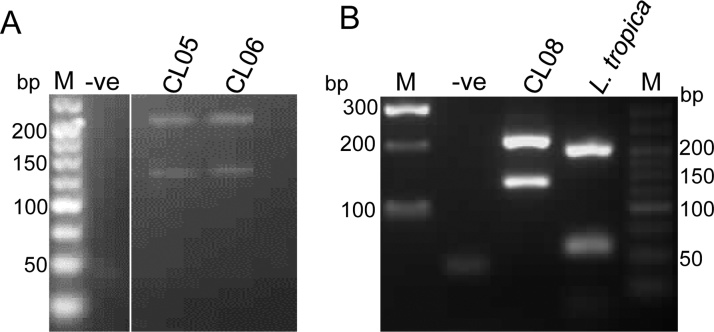
PCR-RFLP identification of *Leishmania* species from cutaneous leishmaniasis lesions. (A) DNA extracted from ATL buffer, CL05 and CL06 produced the RFLP pattern characteristic of *Leishmania major*, with band sizes as described in the text. (B) DNA extracted from culture of the isolate from CL08 (MHOM/SD/2017/ELOBIED) also gave the pattern typical of *L. major*; PCR-RFLP of *L. tropica* was included as control. The *L. major* identification was verified by DNA sequencing (see text).

DNA from FTA cards could not be successfully amplified. For extractions from ATL buffer, PCR-RFLP results were obtained for samples CL05 and CL06, which both had fragment sizes characteristic of *L. major* (203 bp and 132 bp, in accordance with the species-specific pattern described by Schonian et al.) (Schönian et al., 2003) (Figure 4A). From the inoculated cultures, *Leishmania* from CL08 (hereafter named as isolate MHOM/SD/2017/ELOBIED) generated abundant motile promastigotes from which DNA was extracted and used for PCR-RFLP, also yielding the fragment sizes of *L. major* (Figure 4B). MLST of MHOM/SD/2017/ELOBIED produced the expected amplicon sizes (ME, 1653 bp; FH, 1707 bp; PGD, 1440 bp), and DNA sequencing verified *L. major*. Comparison of these sequences with the reference *L. major* genomes revealed 99–100% identity (Supplementary material, Table S3). Nucleotide sequences derived here are available in GenBank under accession numbers MK650465, MK650466, and MK650467. MLST targets were not successfully amplified from CL05 and CL06 DNA extracted from ATL buffer.

## Discussion

### Epidemiology and surveillance

CL principally occurs in populations burdened by poverty, associated with exposure to vectors, poor housing, and population displacement, with a weak immune system, and a lack of economic and health resources (Reithinger et al., 2007, World Health Organization, 2010). Males and females differ in susceptibility to some infectious diseases in an age-dependent manner. A study in Brazil showed a higher incidence of CL caused by New World *Leishmania spp* in males than females, with this disparity increasing with age (Guerra-Silveira and Abad-Franch, 2013). However, for CL it appears that they may be affected equally if both sexes participate in activities/behaviours with high exposure to sand flies (Karimkhani et al., 2016). The presence of infection may depend on immune competence limited by age, impaired by malnutrition or co-infection with HIV, or due to a lack of herd immunity as naïve susceptible individuals accumulate and as populations migrate (World Health Organization, 2010). For *L. major* the incubation period can be less than 4 months, with lesions that may ulcerate and heal within 2–8 months; however, there may be multiple lesions and secondary infections.

Epidemics of CL in Sudan were reported in 1976 in Shendi-Atbara, north of Khartoum (Abdalla and Sherif, 1978), and in 1986 in Khartoum State (el-Safi and Peters, 1991, el-Safi et al., 1991). *L. major* zymodeme LON-1 was identified from the latter epidemic, with a case of diffuse cutaneous leishmaniasis (DCL) reported to be due to this species (Abdel-Hameed et al., 1990). There have been descriptions of uncommon presentations associated with *L. major* (Elamin et al., 2005), and of isolates from cutaneous lesions identified as *L. donovani* (Elamin et al., 2008). Zymodeme MON-74 has also been reported in Sudan (Pratlong et al., 2009), as has *L. major*/HIV co-infection (Mukhtar et al., 2010).

The WHO defines an epidemic threshold as an increase during a similar period that at least doubles compared to 5–10 years of previous data. As described here, from the KDH data, Sudan as a whole has been experiencing increased cases of CL since 2014, and Northern Kordofan has also experienced large increases from 2015, which fulfil the WHO criteria. However, this increase might be due in part to additional reporting and increased awareness. As CL has never been a mandatory notifiable disease in Sudan, MOH data before 2014 are not generally available.

Seasonality, as expected from fluctuating sand fly abundance, was apparent from the occurrence of more CL cases in months with lower temperatures and rainfall, except for Southern Darfur (Figure 1B, C). The wider spread of peaks in Southern Darfur may be due to fewer months of rainfall, variation in reporting patterns, or the presence of conflict in Southern Darfur, which may influence when patients can access health care, or changes in reporting patterns.

In the 1986 CL epidemic in Khartoum State, individuals were affected irrespective of sex, age, ethnic origin, or socio-economic status, suggesting a lack of immunity in the population (el-Safi and Peters, 1991). According to the Northern Kordofan and Southern Darfur MOH data analysed here, fewer cases occurred in those <10 years and >45 years of age compared to the majority of cases between 15 and 44 years old. In Northern Kordofan, these age-related ORs were highly significant, suggesting the following: increased vector exposure among adult agriculturalists, as observed in Ethiopia (Lemma et al., 2015), some protection of infants against exposure, and more common life-long immunity among adults >45 years old. The sex distribution was also skewed in Southern Darfur, with the OR for males more than twice that for females, indicative of a difference in behavioural exposure. However, Southern Darfur State is one of the most populous, including refugees and displaced persons (UNHCR, 2019), and there is likely to be a higher proportion of male soldiers present compared to other regions. Cases in females may also be under-reported if they are less likely to access healthcare facilities.

Several limitations applied to our analyses. In Northern Kordofan and Southern Darfur, there was limited data collection on clinical presentation, treatment, and symptom onset, and no recording of potential risk factors such as immunological status, socio-economic status, and occupation. Due to self-healing of many lesions, as well as poverty and poor access to treatment, many CL patients do not attend healthcare facilities. There were also some inconsistencies between data sources, as in the comparison between 2014 data reported by the WHO (1053 cases for the whole country) and by KDH (1350 cases in this clinic alone). Furthermore, sampling was not focused on seasonal peak.

Thus, it is still difficult to ascertain the true extent of the burden of CL in Sudan, as recognized for African foci generally (Alvar et al., 2012, Sunyoto et al., 2018), and there is an important need for further systematic surveillance and monitoring of CL prevalence and incidence.

### Disease severity and *Leishmania* genetic diversity

Prior to this study, leishmaniasis coordinators in Sudan (including current authors SE-S and AAA) reported both an epidemic increase in cases and more aggressive pathology, unusual for seasonal CL. As *L. major* typically causes more benign self-healing ulcers (World Health Organization, 2010), it was postulated that there was a different disease agent, possibly *L. tropica*, *L. aethiopica*, a novel emergent species, or a new strain of *L. major*.

Here, in the context of a recent CL outbreak in Sudan, PCR-RFLP was used to identify *L. major* in three lesions from three out of 14 patients. Two individuals were young children at the same school. The third was an adult male, from whom MHOM/SD/2017/ELOBIED was isolated; MLST of this isolate revealed >99% sequence identity with reference *L. major* genomic targets (Supplementary material, Table S3).

It was surprising that *L. major* was detected in only three of the 14 individuals with suspected CL. Although CL10, CL11, and CL12 had been diagnosed at other Sudanese health care facilities by microscopy and CL11 via PCR, it was not known whether the lesions had been treated, and two of the four chronic lesions (CL11 and CL12) had secondary bacterial infections. Lesion sampling and storage may have been suboptimal; PCR with FTA card samples has unproven sensitivity. Even for well-trained, skilled technologists, diagnosis by microscopy is demanding. In El Obied Hospital, Northern Kordofan and the HTD in Omdurman, experience in CL lesion sampling diagnoses is limited, and there is a lack of quality control. Thus, false-positive and false-negative results are possible; diagnosis is often made clinically due to a lack of diagnostic resources, and other dermatological conditions may mimic CL.

Other than parasite factors, possible explanations for the reported increase in CL severity include severe malnutrition, increased HIV co-infections, or a population with a more vulnerable genetic background. The current famine declared in South Sudan has brought around a million refugees (UNHCR, 2019). Such population movement is likely to bring an influx of susceptible individuals. In 2014, the spatial sampling survey revealed that 52 of the 184 localities in Sudan had a severe acute malnutrition rating (SAM) of ‘very critical’ (>3%); Southern Darfur was one of three states with the highest SAM recording of >20% (UNICEF, 2014).

## Conclusions and recommendations

Although cases have indeed increased, and we have confirmed that *L. major* is associated with active outbreaks, further surveillance is necessary to uncover the true extent of CL in Sudan. Ideally, simple and standardized surveillance tools could be implemented that promote the systematic recording of essential epidemiological information, such as age, sex, date of symptom onset, and geographical location. Workshops to train relevant personnel could be provided, especially for state MOH Leishmania coordinators, to undertake such basic epidemiological surveys in future studies. Aided by remote communication, perhaps obligatory reporting of these data to designated hubs run jointly by the MOH and the WHO could occur on a regular basis (e.g., monthly).

Similarly, high quality training of laboratory technicians in sampling techniques and the diagnosis of *Leishmania* infection by microscopy could be implemented through workshops. Quality control procedures between state MOH laboratories and specialist Leishmania centres might be feasible to ensure the reliability of the results. Failure to establish quality control may result in further ambiguity of HIS data and line listings that are reported as parasitologically confirmed.

In future research, the development of a reliable point-of-care (POC) rapid diagnostic test (RDT) fulfilling ASSURED criteria (Peeling et al., 2006) would be of great value. Wider phenotypic studies of drug susceptibilities in vitro, virulence in vivo, and comparative genomics of *Leishmania* isolates could be used to investigate the prevalence of drug resistance and question further the emergence of new pathogenic strains.

We recommend that any funding available first be used to standardize surveillance and reporting procedures, sampling techniques, and diagnostic training. This is likely to have a greater and more immediate public health impact on the CL burden. Ideally, a temporary specific task force compiled of experts, leading research institutions, and national Leishmania coordinators could be formed to address these issues and lead to a new control programme.

## Ethics statement

Informed consent was obtained from all participating adults; for children under the age of 18 years, consent was also obtained from their parents or guardians. Verbal explanations and written information sheets in Arabic were provided. The study was approved by the National Health Research Ethics Committee, Federal Ministry of Health, Sudan and the London School of Hygiene and Tropical Medicine, UK. A case report form (CRF) was collected for each sample and unique codes (CL01– CL15) assigned to ensure confidentiality. All subjects and guardians consented to lesion(s) being photographed anonymously. In Northern Kordofan, permission was obtained from the Minister of Health and from community leaders.

## Conflict of interest

None.

## Author contributions

SE-S, MAM, and SC conceived the study. SC, MAM, SE-S, and AAA designed the study. SE-S, AAA, and MDB provided access to field data. AH collected samples. SC collected and analysed field data. SC, TB, and HL performed molecular assays. SC, SE-S, TB, JAW, and MAM drafted the manuscript. MAM, SC, and JAW secured funding.
