# Supplementary material for: Epidemiological and molecular investigation of resurgent cutaneous leishmaniasis in Sudan
Source: Int J Infect Dis. 2019 Nov;88:14–20. doi: 10.1016/j.ijid.2019.08.018 (PMC6838665; doi:10.1016/j.ijid.2019.08.018)
Supplement: Supplementary file 1 [file mmc1.docx]

**Supplementary Table 1.** Primer sequences used for *Leishmania* PCR-RFLP and MLST.

| **Protocol** | **Target** | **Primer** | **Sequence 5’ – 3’** |
| --- | --- | --- | --- |
| PCR-RFLP | ITS-1 | LITSR (For) | CTG GAT CAT TTT CCG ATG |
|  |  | L.5.8S (Rev) | TGA TAC CAC TTA TCG CAC TT |
| MLST | *ME* | For | CGC AAC CGC TTC ACC AA TAA GGG C |
|  |  | Rev | CAA CTC CTT CTC CAG GTA GTA GTT |
| MLST | *FH* | For | AGC GTC TTG TGT TTC CCA |
|  |  | Rev | GAG CCC GTG TAA GGA GGC |
| MLST | *PGD* | For | GAA CGA ATC CCT TAT TCT CYA TG |
|  |  | Rev | GGA ACC GGT TGA GCG GC |

*Leishmania* species identification. ITS1-*Hae*III PCR-RFLP analysis was applied for species identification. Amplification reactions were performed in a total volume of 20 µl, comprising of 1 x NH_4_ reaction buffer with 1.5 mM MgCl_2_ (Bioline, UK), 200 μM dNTPs (New England Biolabs, UK), 10 pmol of each primer and 1 U BioTaq DNA polymerase (Bioline). For FTA cards, a 3 mm diameter punch section was added directly into the PCR mix; for DNA extracted from ATL buffer and culture 1 μl was used as template. Amplification conditions were: 1 cycle of 94°C for 2 mins; 33 cycles of 94°C for 30 secs, 53°C for 30 secs, 72°C for 1 min; and 1 cycle of 72°C for 10 mins. Ten microlitres of the PCR product were digested with *Hae*III (New England Biolabs) and run on 3% agarose gels.

*Leishmania* multilocus sequence typing (MLST). Genomic targets for amplification were: *ME*, encoding cytosolic NADP-malic enzyme (EC1.1.1.40); *FH*, fumarate hydratase (EC 4.2.1.2); *PGD*, 6-phosphogluconate dehydrogenase (EC 1.1.1.44). Composition of amplification reactions were as above, with amplification: 1 cycle of 95°C for 2 mins; 30 cycles of 95°C for 1 minute, 50°C (*me*) / 58°C (*fh*) / 60°C (*pgd*) for 1 minute, 72°C for 90 seconds, and 1 cycle of 72°C for 10 minutes.

**Supplementary Table 2.** Age and sex OR of CL cases reported by Southern Darfur MOH and Northern Kordofan line listings from two hospitals.

| State |  | Cases | Population (000s)^a^ | OR [95% CI] | *P* value^b^ |
| --- | --- | --- | --- | --- | --- |
| Southern Darfur  Jan 2016-Aug 2017 |  |  |  |  |  |
| Age groups (years) | >45 | 27 | 570·0 | 1·0 |  |
|  | 35-44 | 30 | 455·4 | 1·39 [0.80-2·43] | 0.2117 |
|  | 20-34 | 77 | 1,169·3 | 1·39 [0·89-2·24] | 0.1390 |
|  | 0-19 | 201 | 2,893·7 | 1·47 [0·98-2·28] | 0.0602 |
|  | Not reported | 7 | - |  |  |
|  | TOTAL | 342 | 5,088·4 |  |  |
| Sex | Female | 95 | 2,431·0 | 1·0 |  |
|  | Male | 247 | 2,657·4 | 2·38 [1·87-3·05] | <0·0001 |
|  | TOTAL | 342 | 5,088·4 |  |  |
|  |  |  |  |  |  |
| Northern Kordofan  July 2016-July 2017 |  |  |  |  |  |
| Age groups (years) | >45 | 20 | 430·2 | 1·0 |  |
|  | 35-44 | 34 | 280·9 | 2·60 [1·46-4·77] | 0.0004 |
|  | 20-34 | 66 | 677·5 | 2·10 [1·25-3·65] | 0.0030 |
|  | 0-19 | 91 | 1,751·5 | 1·12 [0·68-1·91] | 0.6525 |
|  | Not reported | 4 | - |  |  |
|  | TOTAL | 215 | 3,140·2 |  |  |
| Sex | Female | 105 | 1,629·8 | 1·0 |  |
|  | Male | 108 | 1,510·4 | 1·11 [0·84-1·47] | 0·4467 |
|  | Not reported | 2 | - | - | - |
|  | TOTAL | 215 | 3,140·2 |  |  |

a = Projections for 2016.

b = chi^2^ test

**Supplementary Table 3.** MLST sequence matches of MHOM/SD/2017/ELOBIED with reference genomes.

|  | **ME** | **FH** | **PGD** |
| --- | --- | --- | --- |
| **Friedlin** | 1646/1653 bp | 1707/1707 bp | 1431/1440 bp |
| **LV39** | 1646/1653 bp | 1707/1707 bp | 1432/1440 bp |
| **SD75** | 1647/1653 bp | 1707/1707 bp | 1435/1440 bp |
